# Adenoid Hypertrophy and Orthodontic Complications: An Assessment of Parental Knowledge in Saudi Arabia

**DOI:** 10.7759/cureus.41692

**Published:** 2023-07-11

**Authors:** Huda A Alzahrani, Raghad O Alkhaldi, Demah H Alsufyani, Shatha A Alghamdi, Tahani A Althobity, Yahya A Fageeh

**Affiliations:** 1 Medicine and Surgery, College of Medicine, Taif University, Taif, SAU; 2 Otolaryngology, Head and Neck Surgery, College of Medicine, Taif University, Taif, SAU

**Keywords:** upper airway obstruction, mouth breathing, adenoidectomy, orthodontic complications, adenoid hypertrophy

## Abstract

Background

Adenoid hypertrophy is a common condition that can cause upper airway obstruction in children and adolescents, leading to various complications, including dental and maxillofacial abnormalities. However, parents may have limited knowledge of the orthodontic complications associated with this condition.

Objective

This study aimed to assess the knowledge and attitude of parents toward the orthodontic complications of untreated adenoid hypertrophy and to promote their awareness about this problem.

Method

This descriptive cross-sectional study involved 824 parents from all regions of Saudi Arabia. An online questionnaire was used to collect data on parents' demographics, their children's information, and their general knowledge about adenoid hypertrophy, including its symptoms, complications, and treatment.

Results

The study included 824 parents with a mean age of 36.78 ± 10.87 years, 73.2% of whom were women. Overall, only 6.2% (51.1 parents) had a good level of knowledge about adenoid hypertrophy.

Conclusion

The study highlights the importance of promoting awareness and educating parents about the orthodontic complications associated with adenoid hypertrophy. Greater awareness and understanding can help parents make better decisions for their children's health and well-being.

## Introduction

Adenoid hypertrophy is a common disorder associated with upper airway obstruction, with a reported prevalence of 34.46% among children and adolescents, according to a recent meta-analysis [1]. This condition is considered one of the most common causes of upper airway obstruction [2].

Adenoid hypertrophy can have infectious and non-infectious causes. Infectious causes are more common, driven by viral and bacterial pathogens [3,4].

The symptoms of adenoid hypertrophy vary in children depending on the obstructed structure, with the most common symptoms including rhinorrhea, difficulty breathing through the nose, persistent cough, postnasal drip, snoring, breathing problems during sleep, and difficulty swallowing. When the adenoid is enlarged and blocks the nasopharynx, it can lead to eustachian tube dysfunction and otitis media with effusion. In addition, the patient may develop sinusitis due to nasopharyngeal obstruction and associated infections [5].

Adenoidal obstruction can be graded into four levels. Grade I indicates that the adenoid tissue blocks 0% to 25% of the posterior choana. while grade II indicates 26% to 50% obstruction. Grade III indicates 51% to 75% obstruction, and grade IV indicates 76% to 100% obstruction [6].

Adenoid hypertrophy can result in various health problems. These complications include obstructive sleep apnea, marked by repeated breathing pauses during sleep and leading to poor sleep quality and daytime fatigue. In children, adenoid hypertrophy can also have a negative impact on academic performance and behavior. Additionally, chronic mouth breathing, often caused by adenoid hypertrophy, can affect the growth of the craniofacial bones, leading to malocclusion and dental abnormalities over time [7-9].

Various methods are used to categorize malocclusions, but the most commonly employed one is Angle's classification system (1898). It is based on the relationship between the mesial buccal cusp of the maxillary first molar and the buccal groove of the mandibular first molar, and it divides malocclusions into three classes: class I, where the cusp fits into the groove; class II, where the cusp is distal to the groove; and class III, where the cusp is mesial to the groove. Patients with adenoid hypertrophy frequently exhibit a class II malocclusion, which is characterized by several dental features such as crowded teeth, retroclined mandibular incisors, a narrow upper alveolus, a hypoplastic maxilla, a high-arched palate, an anterior tongue position, and prominent upper teeth. Additionally, patients with adenoid hypertrophy may display certain external features, including an open-mouth posture, an elongated face, pinched nostrils, a short upper lip, an increased lower lip, a steep angle of the mandible, mandibular retrognathism, and a vacant expression [10,11].

Adenoidectomy is the recommended and most effective treatment for children with complications from adenoid hypertrophy. Therefore, it is essential to intervene early to minimize the likelihood of associated complications. However, the success of early intervention is dependent on parents' awareness and knowledge regarding adenoid hypertrophy and its potential complications. Regrettably, there is limited research available on this subject. Thus, this study aims to evaluate parents' awareness and attitude toward the orthodontic problems that might arise from untreated adenoid hypertrophy and to increase awareness about this issue.

## Materials and methods

Study design

A descriptive cross-sectional study design was used. The study was conducted in Saudi Arabia from September 2021 to December 2022. The study population included parents living in all regions of Saudi Arabia. We excluded nonparents, parents with children > 12 years old, and those who refused to participate in the study.

An online questionnaire was distributed to 824 parents by sending a link to their phone numbers. They were invited to participate in the survey, and the purpose of the study was explained to them before they participated. The questionnaire consisted of open and closed questions that had been formulated based on several previous studies [12-17].

The questionnaire

The questionnaire was developed and revised by two otolaryngologists. The questionnaire aimed to assess parents' knowledge of adenoid hypertrophy, which includes its symptoms, complications, and treatment. The questionnaire consisted of four sections. The first section gathered demographic information about participants, including gender, age, education level, residence, and income. The second section included questions about the children's data, such as the number of children, if they were diagnosed with adenoid hypertrophy, their gender and age, and whether they had visited an ENT clinic or dental clinic. The third section assessed general knowledge about adenoid hypertrophy and its symptoms. We asked them about symptoms of adenoid hypertrophy like snoring, mouth breathing, restless sleep, bedwetting, hyperactivity, lack of attention, aggressive behavior, headache, daytime somnolence, and hearing and speech difficulties. The fourth section focused on the participants' knowledge about orthodontic complications of adenoid hypertrophy, including dental decay, a gummy smile, crowded teeth, an elongated face, a flat nose, retrognathism, a vacant expression, prominent upper teeth, and a high-arched palate.

To assess the level of knowledge, a score of "1" was given for correct answers, while "0" was given for incorrect answers or "I don't know." Individuals who answered 80% or more of the questions correctly were deemed to have good knowledge, while those who scored less than 80% were considered to have poor knowledge [18,19].

Data analysis

The IBM Statistical Package for the Social Sciences (SPSS) statistical program version 26 (Armonk, NY: IBM Corp.) was used for data analysis. The Chi-squared test (χ2) was used to examine the relationship between variables, and the Mann-Whitney test was used for non-parametric variables. Quantitative data were presented as mean ± SD, and correlation analysis was performed using Spearman's test. A p-value of less than 0.05 was considered statistically significant.

Ethical considerations

The study obtained ethical approval from the Research Ethical Committee at Taif University, Taif, Kingdom of Saudi Arabia (No. 43-058). Questionnaires were anonymous to ensure confidentiality.

## Results

The study enrolled participants with an average age of 37 years, most of whom were mothers and had completed university education. The majority of participants had two or more children with an average age of seven years, and approximately 28% had at least one child diagnosed with adenoid hypertrophy (Table 1).

**Table 1:** The distribution of study participants according to their demographic characteristics and their children's data (N=824) SR: Saudi Riyal

Variable	No. (%)
Age (years)	36.78 ± 10.87
Gender	
Female	603 (73.2)
Male	221 (26.8)
Educational level	
Elementary school	33 (4)
Secondary school	126 (15.3)
University	596 (72.3)
Postgraduate	69 (8.4)
Residence	
Rural	71 (8.6)
Urban	753 (91.4)
Monthly family income (SR)	
Low (less than 5,000 SR)	89 (10.8)
Moderate (5,000 SR –15,000 SR)	530 (64.3)
High (more than 15,000 SR)	205 (24.9)
How many children do you have?	
One	168 (20.4)
Two	215 (26.1)
Three	180 (21.8)
Four	142 (17.2)
Five or more	119 (14.4)
What is your child's gender?	
Girl	358 (43.4)
Boy	466 (56.6)
Child's age (years)	7.08 ± 3.17
Has your child ever visited the ENT clinic?	
No	456 (55.3)
Yes	368 (44.7)
Has your child ever visited the dental clinic?	
No	479 (58.1)
Yes	345 (41.9)
Has any one of your children been diagnosed with adenoid hypertrophy?	
No	596 (72.3)
Yes	228 (27.7)

**Figure 1:**
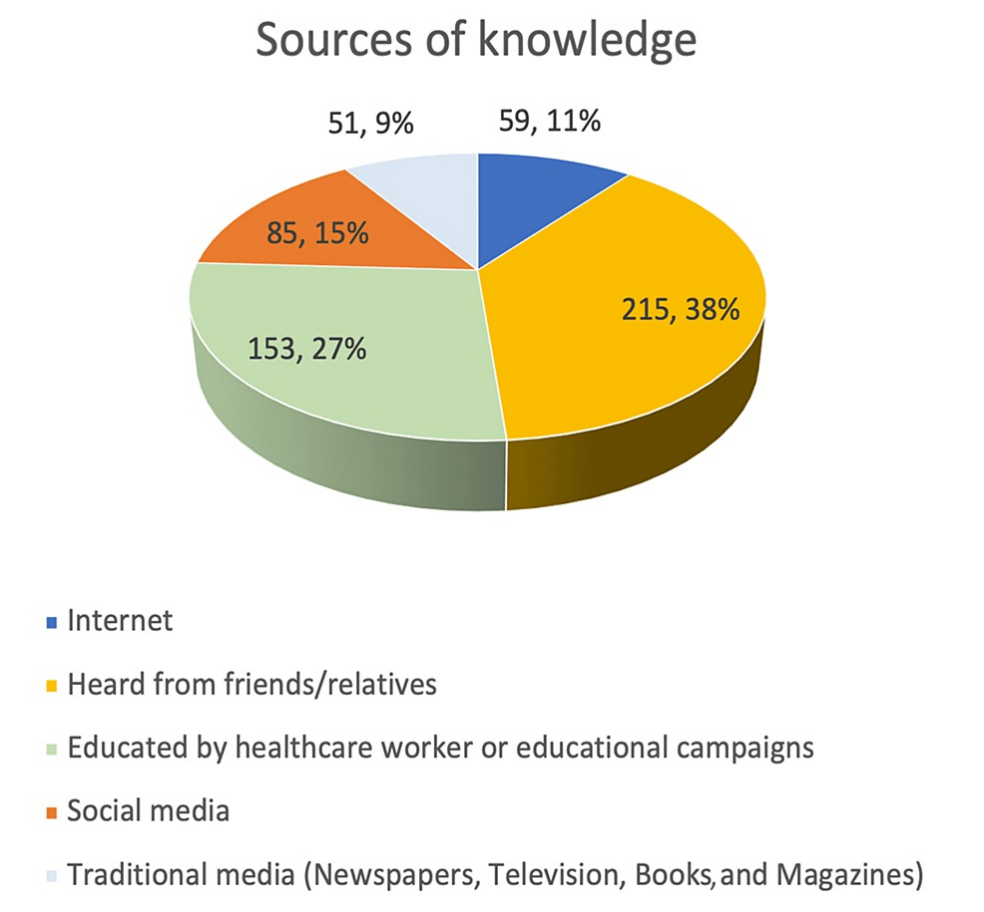
The distribution of study participants according to their knowledge about adenoid hypertrophy in children and their /sources of knowledge (N=824)

The study revealed that a significant proportion of participants (68.6%) were unaware of this condition in children, and their primary source of information was friends or family members (Figure 1).

According to the participant's responses, the most common symptoms of adenoid hypertrophy include snoring, mouth breathing, sleeping with an open mouth, and difficulty speaking (Table 2).

**Table 2:** The frequency and percentage distribution of participants' correct answers about symptoms and complications items regarding adenoid hypertrophy (N=824)

Variable	No. (%)
Symptoms of adenoid hypertrophy	
Hearing difficulty	194 (23.5)
Speaking difficulty	531 (64.4)
Hyperactivity	66 (8)
Lack of attention	87 (10.6)
Aggressive behavior	51 (6.2)
Headache	350 (42.5)
Bedwetting	77 (9.3)
Dry mouth	392 (47.6)
Thirsty awakening at nights	336 (40.8)
Negative effects on school performance	60 (7.3)
Restless sleep	115 (14)
Daytime somnolence	84 (10.2)
Open mouth during sleeping	627 (76.1)
Snoring	676 (82)
Mouth breathing	660 (80.1)
Choking or gasping while asleep	497 (60.3)
Inflammation of the nasal cavity and sinuses	241 (29.2)
Recurrent ear infections	348 (42.3)

Furthermore, a majority of participants (67.1%) were aware that bad breath could be a complication of this condition, while 64.1% knew that it could result in a narrower airway.

Regarding orthodontic complications resulting from adenoid hypertrophy and mouth breathing, tooth decay was recognized by only 22.1% of participants, while 34.6% knew about gum diseases. Furthermore, 39.1% knew the risk of developing a high-arched palate, while fewer participants were aware of other potential abnormalities such as crowded teeth (31.6%), retrognathism (31.4%), and prominent upper incisors (26.5%) (Table 3).

**Table 3:** The frequency and percentage distribution of participants' correct answers about the orthodontic complications of adenoid hypertrophy (N=824)

Which of the following do you think is a complication of adenoid hypertrophy?	
Tooth decay	181 (22.1)
Gum diseases like gingivitis, short upper tooth and lips (gummy smiles)	285 (34.6)
Crowded teeth (narrow upper alveolus)	260 (31.6)
Bad breath	553 (67.1)
Long and narrow face	184 (22.3)
Small and flat nose	201 (29.4)
Set back lower jaw (retrognathism) and a steeper angle of mandible	259 (31.4)
Poor definition of cheekbones (vacant expression)	111 (13.5)
Smaller airway	528 (64.1)
Prominent upper teeth	218 (26.5)
Retroclined upper incisors	136 (16.5)
High-arched palate	322 (39.1)

**Figure 2:**
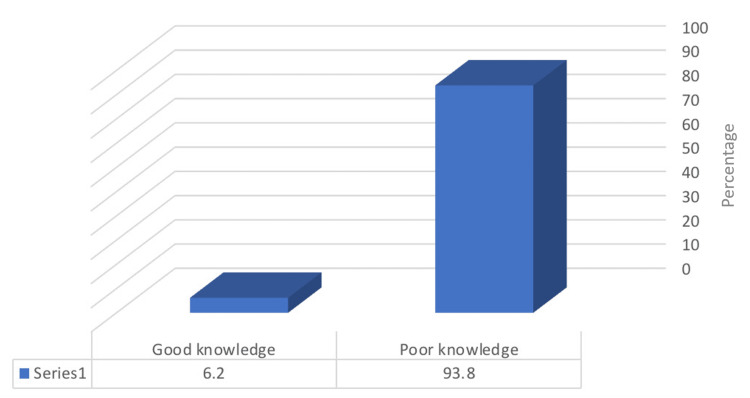
The percentage distribution of the participants according to their level of knowledge about adenoid hypertrophy (N=824)

Most participants (77.5%) believed that hospital visits are necessary if their child exhibits any of the symptoms associated with adenoid hypertrophy. However, only 47.8% of participants knew that untreated adenoid hypertrophy and mouth breathing could lead to dental problems, while 37.1% knew this condition could affect the development of the jaws and face. Nearly all participants (90.8%) believed that children with adenoid hypertrophy require treatment, and the majority (92%) agreed that adenoidectomy is an effective treatment for obstructive sleep apnea. Additionally, 88.1% of participants perceived adenoidectomy as a helpful intervention to correct related orthodontic abnormalities. Remarkably, only a small proportion (6.2%) of participants had good knowledge about adenoid hypertrophy (Figure 2).

Finally, the study did not identify a significant relationship between the participants' knowledge level about adenoid hypertrophy and their demographic characteristics or their children's data (Table 4).

**Table 4:** The relationship between participants' knowledge about adenoid hypertrophy and their demographic characteristics and children's data (N=824)

Variable	Knowledge level		χ2	p-value
	Poor No. (%)	Good No. (%)		
Gender				
Female	569 (94.4)	34 (5.6)	1.17	0.278
Male	204 (92.3)	17 (7.7)		
Educational level				
Elementary school	33 (100)	0 (0.0)	5.23	0.155
Secondary school	121 (96)	5 (4)		
University	557 (93.5)	39 (6.5)		
Postgraduate	62 (89.9)	7 (10.1)		
Residence				
Rural	63 (88.7)	8 (11.3)	3.45	0.063
Urban	710 (94.3)	43 (5.7)		
Family income (SR)				
Low (less than 5,000 SR)	84 (94.4)	12 (5.9)	0.13	0.934
Moderate (5,000 SR -15,000 SR)	496 (93.6)	34 (6.4)		
High ( more than 15,000 SR)	193 (94.1)	12 (5.9)		
How many children do you have?				
One	157 (93.5)	11 (6.5)	0.99	0.911
Two	200 (93)	15 (7)		
Three	168 (93.3)	12 (6.7)		
Four	135 (95.1)	7 (4.9)		
Five or more	113 (95)	6 (5)		
What is your child's gender?				
Girl	339 (94.7)	19 (5.3)	0.84	0.84
Boy	434 (93.1)	32 (6.9)		
Has your child ever visited the ENT clinic?				
Yes	424 (93)	32 (7)	1.2	0.272
No	349 (94.8)	19 (5.2)		
Has your child ever visited the dental clinic?				
Yes	445 (92.9)	34 (7.1)	1.62	0.202
No	328 (95.1)	17 (4.9)		
Has any one of your children been diagnosed with adenoid hypertrophy?				
No	557 (93.5)	39 (6.5)	0.46	0.496
Yes	216 (94.7)	12 (5.3)		

## Discussion

This study aimed to evaluate parents' awareness and attitudes regarding the orthodontic complications of untreated adenoid hypertrophy and to improve their knowledge about this condition.

The study found that the most common symptoms of adenoid hypertrophy were snoring, mouth breathing, an open mouth during sleep, and difficulty speaking. Adenoid hypertrophy primarily affects nasal breathing and might cause pauses in breathing during sleep when associated with enlarged palatine tonsils [20].

Adenoid hypertrophy can cause persistent middle ear effusion, which may lead to conductive hearing loss resulting in speech, language, and learning difficulties [21,22].

Additionally, adenoid hypertrophy increases the risk of sleep-disordered breathing and sleep apnea, resulting in behavioral problems, pulmonary hypertension, and psychiatric disorders such as depression and attention deficit hyperactivity disorder (ADHD) [23].

In our study, we detected insufficient parental knowledge regarding the orthodontic complications related to untreated adenoid hypertrophy.

The study identified insufficient parental knowledge regarding orthodontic complications related to untreated adenoid hypertrophy. Less than half of the parents reported that untreated adenoid hypertrophy and mouth breathing could cause dental problems. Further, only about a third of parents knew that untreated adenoid hypertrophy and mouth breathing could affect the development of the jaws and face. Adenoid hypertrophy can result in deformities in the dental arc and facial skeleton due to chronic mouth breathing, as it causes upper airway obstruction. The "adenoid face" is characterized by upper lip incompetence, a retroposition of the hyoid bone, a narrow upper dental arch, a retroposition of mandibular incisors, increased anterior face height, a narrow or "V"-shaped maxillary arch, an increased mandibular plane angle, and a posterior-rotated mandible compared to healthy controls [24-26].

The majority of parents believed that children with adenoid hypertrophy needed medical attention. They also agreed that surgical intervention (adenoidectomy) effectively treats obstructive sleep apnea and can correct related orthodontic complications. Notably, most cases of obstructive sleep apnea in children are caused by adenotonsillar hypertrophy, which makes adenotonsillectomy the primary treatment option [27, 28].

The findings of this study emphasize the importance of promoting awareness and educating parents about the orthodontic complications associated with adenoid hypertrophy, as well as the significance of early diagnosis and treatment to prevent its complications. Healthcare professionals have a crucial role to play in educating parents about the importance of early diagnosis and treatment of adenoid hypertrophy. Also, this education can be facilitated through education campaigns targeting parents.

## Conclusions

The study found that parents have limited knowledge about the orthodontic abnormalities associated with adenoid hypertrophy. Therefore, it is essential to educate and raise parental awareness about adenoid hypertrophy and its potential complications, particularly its effects on dental and maxillofacial development. This education can be facilitated through healthcare providers and health education campaigns. Increased awareness and understanding of adenoid hypertrophy and its complications can empower parents to make informed decisions about their children's health and improve overall health outcomes.
